# Lycopene prevents non-alcoholic fatty liver disease through regulating hepatic NF-κB/NLRP3 inflammasome pathway and intestinal microbiota in mice fed with high-fat and high-fructose diet

**DOI:** 10.3389/fnut.2023.1120254

**Published:** 2023-03-23

**Authors:** Xiang Gao, Xia Zhao, Min Liu, Huimin Zhao, Yongye Sun

**Affiliations:** ^1^Institute of Nutrition and Health, College of Public Health, Qingdao University, Qingdao, China; ^2^College of Life Sciences, Qingdao University, Qingdao, China; ^3^Department of Pediatric Dentistry, Qingdao Stomatological Hospital Affiliated to Qingdao University, Qingdao, China; ^4^Department of Diet and Nutrition, Shandong Provincial Chronic Disease Hospital, Qingdao, China

**Keywords:** lycopene, NLRP3, nonalcoholic fatty liver disease, intestinal microbiota, mice

## Abstract

Lycopene (LY) belongs to carotenoids and is abundant in red fruits and vegetables. Several previous studies suggested that LY is beneficial for ameliorating non-alcoholic fatty liver disease (NAFLD), while the potential mechanisms are unclear. The present study aimed to clarify the potential mechanisms of LY in preventing NAFLD *via* exploring the hepatic NF-κB/NLRP3 inflammasome pathway and intestinal microbiota composition in high-fat and high-fructose diet (HFFD)-fed mice. Fifty eight-week-old male C57BL/6J mice were randomly assigned into 5 groups: Normal control group (NC); HFFD group; HFFD with low dose of lycopene group (LLY, 20 mg/kg/d); HFFD with high dose of lycopene group (HLY, 60 mg/kg/d) and HFFD with resveratrol group (RSV, 50 mg/kg/d, positive control). After 8 weeks, feces were collected and the 12 h fasted mice were sacrificed to acquire tissues and blood for parameters measurement. The results showed that the mice in LLY, HLY and RSV groups had significantly lower body weight gain, weight of white adipose tissue, serum levels of high density lipoprotein-cholesterol (HDL-C), low density lipoprotein-cholesterol (LDL-C), lipopolysaccharide (LPS), alanine aminotransferase (ALT), and hepatic concentrations of triglyceride (TG) and interleukin-6 (IL-6) than that in the HFFD group (*p* < 0.05). HLY and RSV groups also displayed lower serum levels of TG, total cholesterol (TC) and hepatic levels of tumor necrosis factor-α (TNF-α) than the HFFD group (*p* < 0.05). Liver protein expressions of NLRP3, Pro-Caspase-1, Caspase-1 and NF-κB were lower in the LLY, HLY and RSV groups than those in the HFFD group (*p* < 0.05). The feces of LY -treated mice had higher relative levels of SCFAs producing bacteria *Allobaculum* and lower destructive bacteria, including *Firmicutes*, *Lachnospiraceae_NK4A136_group*, *Desulfovibrio*, and *Alistipes* over the HFFD group (*p* < 0.05). RSV group also displayed lower fecal levels of *Lachnospiraceae_NK4A136_group*, *Desulfovibrio*, and *Alistipes* than the HFFD group (*p* < 0.05). In conclusion, LY might prevent NAFLD by suppressing hepatic NF-κB/NLRP3 inflammasome pathway and attenuating gut microbiota dysbiosis.

## 1. Introduction

Non-alcoholic fatty liver disease (NAFLD) is defined by macrovesicular steatosis in more than 5% of hepatocytes, in the absence of alcohol or drugs (1). Currently, NAFLD is one of the most common chronic liver diseases worldwide, with a 20–30% of prevalence in general population (2). NAFLD may further develop into cirrhosis and hepatocellular carcinoma (HCC), and is closely related with metabolic syndrome, type 2 diabetes, cardiovascular diseases, and other metabolic diseases (3). Nowadays, no effective medicine for NAFLD is available. Accordingly, there is an urgent need to better understand the pathogenesis of NAFLD and discover novel therapeutic agents.

The pathogenesis of NAFLD is complex and “multiple hits” has been recognized as the most accepted theory. Cross-talk between host genetics and environmental factors leads to the occurrence of parallel multiple hits, such as inflammation, oxidative stress, lipid peroxidation, mitochondrial dysfunction, etc. Numerous studies proved that chronic inflammation is a pivotal trigger for the initiation and progression of NAFLD (4). NAFLD is related with massive release of pro-inflammatory factors, such as interleukin-6 (IL-6), tumor necrosis factor-α (TNF-α) and interleukin-1β (IL-1β) (5). Recently, the NOD-like receptor family pyrin domain containing 3 (NLRP3) inflammasome is recognized to drive the chronic inflammation and plays a key role in the development of NAFLD (6). During NAFLD, multiple danger signals activate the nuclear factor kappa B (NF-κB) and further trigger the activation of NLRP3 inflammasome pathway, which promotes the maturation of caspase-1 and leads to the production of pro-inflammatory cytokines IL-1β and IL-18. The excessive release of IL-1β and IL-18 aggravates chronic inflammation and mediates hepatic steatosis and other metabolic disorders (7). Blockade of NLRP3 inflammasome reversed liver inflammation and hepatic lipids deposition in choline deficiency (8) or HFD induced (9) NAFLD mice.

Recently, increasing literatures indicated that gut microbiota plays an essential role in NAFLD development and evolution (10). A variety of microbial communities are located in the intestinal tract that provide various benefits for maintenance of host health and are affected by numerous factors, like diet, genetics and exposure to drugs, etc. (10). A series of studies have reported gut microbial dysbiosis in patients or rodents with NAFLD (11). Germ-free (GF) animals are resistant to NAFLD induced by high-fat diet or western diet (12) and fecal microbiota transplantation reversed hepatic steatosis in HFD fed mice (13). Compelling evidences have indicated that intestinal flora generates a variety of substances that interact with liver cells of the host through the portal vein and involves in the initiation of hepatic inflammation and development of NAFLD (14). Lipopolysaccharide (LPS) is a component of the cell membrane of gram-negative bacteria and can trigger hepatic inflammation by binding to toll-like receptor 4 (TLR4), activating inflammatory pathways and the NLRP3 inflammasome in liver (15). Short-chain fatty acids (SCFAs), such as acetate, propionate, and butyrate etc., are generated from the gut microbial fermentation of dietary fiber and have beneficial effects for maintaining gut barrier, inhibition LPS-driven inflammatory responses and alleviating NAFLD (16). Elevated fecal levels of LPS and decreased that of SCFAs were observed in subjects or rodents with NAFLD (17).

Lycopene (LY) belongs to carotenoids and is abundant in red fruits and vegetables such as tomatoes, watermelons, guavas, and pink grapefruits (18). Numerous biological activities of LY were identified, like antioxidant, anticancer, anti-inflammation, cardiovascular and neural protective effects, etc. (19). Some animal studies have reported LY supplementation ameliorated hepatic steatosis and decreased serum alanine aminotransferase (ALT) and aspartate aminotransferase (AST) levels in obese rodents (20), indicating the protective effect of LY on NAFLD. However, the potential mechanisms are largely unknown. To date, no study has investigated the roles of NLRP3 inflammasome and gut microbiota in the protective effect of LY on NAFLD.

In this study, we aimed to explore the effect of LY on the hepatic NF-κB/NLRP3 inflammasome pathway and intestinal microbiota composition, and examine their relationships with NAFLD in mice. The findings will provide further evidence for the regulative effects and mechanisms of LY on NAFLD.

## 2. Materials and methods

### 2.1. Protocol of animal study

The laboratory animal welfare ethics committee of Qingdao University approved this study (20201008C5720201209081). Fifty eight-week-old male C57BL/6J mice (SCXK 2016–0006) were provided by Vital River Laboratory Animal Center (Beijing, China). They were housed in standard laboratory cages under a standard condition (temperature: 21 ± 2°C, humidity: 50–60% and 12 h- light/dark cycle). After a adaption period, the mice were randomly assigned into 5 groups (n = 10): Normal control group (NC), mice fed with a normal chow diet (Research Diets: D12450H; 10% calories from fat) + saline solution; (2) High-fat and high-fructose diet group (HFFD), mice fed with a HFD (Diet serial number: D12451; 45% calories from fat) + 10% fructose solution; (3) Low dose lycopene group (LLY), mice fed with a HFD + 10% fructose solution +20 mg/kg/d lycopene (oral gavage); (4) High dose lycopene group (HLY), mice fed with a HFD + 10% fructose solution +60 mg/kg/d lycopene (oral gavage); (5) Resveratrol positive control group, mice fed with a HFD + 10% fructose solution +50 mg/kg/d resveratrol (oral gavage). The lycopege (10%) was pruchased from Fine (Guangzhou) Boitechnology Co., Ltd. and the resveratrol (98%) was provided by Xi’an Victory Biochemical Technology Co., Ltd. After 8 weeks, feces were acquired and subsequently the 12 h-fasted mice were anesthetized by intraperitoneal injection of 500 mg/kg chloral hydrate and sacrificed to separate tissues and serum.

### 2.2. Measurement of biochemical parameters

Serum levels of fasting blood glucose (FBG), total cholesterol (TC), triglyceride (TG), low density lipoprotein-cholesterol (LDL-C), high-density lipoprotein cholesterol (HDL-C), ALT and AST were detected with an automatic analyser (Hitachi Instruments Co. Ltd., Tokyo, Japan). ELISA kits (Bioscience, Inc., Thermo, United States) were utilized to evaluate serum insulin and hepatic TNF-α and IL-6. Serum LPS was detected according to ELISA kit instructions (Jingmei, Jiangsu, China). TG and TC in liver were measured with commercial kits. Homeostasis model assessment-insulin resistance (HOMA-IR) (21), which equals to FBG times insulin divided by 22.5 was calculated.

### 2.3. Histological analysis of the liver

The rapidly removed liver of the mice were fixed in a 10% formaldehyde solution for 24 h. Hema-toxylin–eosin (H&E) staining process was referenced to our previous publication (22). The microscopic structures were observed on a light microscope (Olympus, Tokyo, Japan; 400×). NAFLD activity score (NAS) was determined according to previous literature based on the grade of steatosis, inflammation and balloning (23).

### 2.4. Western blot analysis

The livers (30 mg) were homogenized in cold RIPA lysis buffer and centrifuged to collect supernatant. The extracted total proteins were qualified by a BCA Protein Quantitation Kit and then loaded onto 10% SDS-PAGE followed by transferring to polyvinylidene fluoride membranes, which were subsequently blocked with skim milk, and incubated overnight with different primary antibodies (Abcam Bioscience, New York, America): β-actin (1:5000), TLR-4 (1:1000), NF-κB (1:1000), NLRP3 (1:1000), Caspase-1 (1:1000), IL-1β (1:1000). Afterwards, the membranes were washed and incubated with a secondary antibody (1:7500) for 1.5 h. The protein bands were observed by autoradiography using an enhanced chemiluminescence (ECL) localization reagent and quantified by Tanon GIS analysis.

### 2.5. Real-time quantitative PCR analysis

Total RNA was extracted from liver using TRIzol reagent. Purity and quantity of RNA were assessed and 1 μg RNA was reversely transcribed into cDNA. The RT- PCR was carried out by SYBR Premix Ex Taq fluorescent quantitative PCR (Eppendorf, America). The PCR cycling conditions were: 1 cycle of 95°C for 30 s, 40 cycles of 95°C for 5 s, 60°C for 32 s. All the primer are listed in Supplementary Table 1.

### 2.6. Microbial diversity analysis

The bacterial DNA was extracted from feces and sequenced by BmK Biotechnology Co., Ltd. (Qingdao, China). PCR amplification was performed on the variable region 3–4 (V3–V4), (24). The data were analyzed online.^1^,^2^ Alpha diversity and Beta diversity were calculated. Relative abundance of bacteria was assessed.

### 2.7. Statistical analysis

Data are shown as mean ± standard deviation (SD). The normal distribution of the data was confirmed by Shapiro -Wilk test. The mean differences among groups were determined by one-way analysis of variance (ANOVA) followed by LSD *post hoc* test. Spearman correlation analysis was applied to analyze the associations of the bacteria with the NAFLD-related indices. Statistically significant was considered as *p* < 0.05. SPSS 21.0 software was used for all the analyses. Graphpad Prism 8.0 and Figdraw^3^ were used for drawing of figures.

## 3. Results

### 3.1. Effect of lycopene on the growth parameters and serum biochemicals

**Figure 1:**
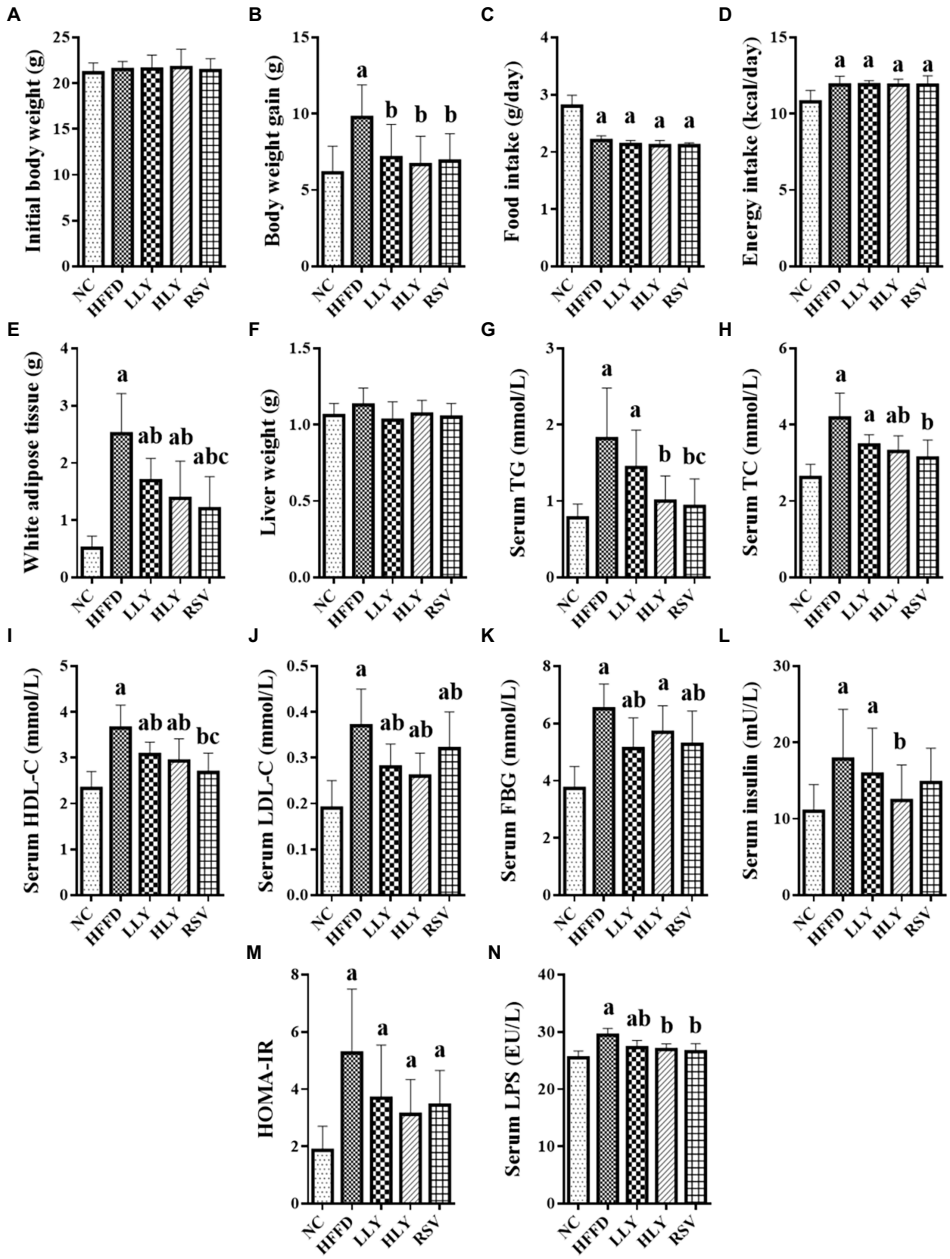
Effects of Lycopene on growth parameters and serum biochemicals in the HFFD-fed mice. **(A)** Initial body weight; **(B)** Body weight gain; **(C)** Daily food intake; **(D)** Daily energy intake; **(E)** Weight of white adipose tissue; **(F)** Liver weight; **(G)** Serum levels of TG; **(H)** Serum levels of TC; **(I)** Serum levels of HDL-C; **(J)** Serum levels of LDL-C; **(K)** Serum levels of FBG; **(L)** Serum levels of Insulin; **(M)** HOMA-IR; **(N)** Serum levels of LPS; White adipose tissue, indicating the perirenal and epididymal adipose tissue; ^a^*p* < 0.05, ^b^*p* < 0.05, ^c^*p* < 0.05 compared with the NC, HFFD and LLY groups, respectively.

As shown in Figure 1, body weight gain and the weight of white adipose tissue (WAT) of the mice in HFFD group were higher than those in the NC group after 8 weeks feedings (*p* < 0.05 for both). LY and RSV treatment mice displayed lower body weight gain and the weight of WAT compared to the HFFD-fed mice (*p* < 0.05). The food intake was lower, while the energy intake was higher in the HFFD-fed groups than the NC group (*p* < 0.05). There was no difference on the initial body weight and liver weight among the experimental groups.

HFFD led to significant elevated serum levels of TG, TC, HDL-C, LDL-C, FBG, insulin, LPS and HOMA-IR index (*p* < 0.05). LY supplemented mice had lower serum levels of HDL-C, LDL-C and LPS than the HFFD group (*p* < 0.05). HLY group also showed lower serum levels of TG, TC and insulin compared to the HFFD group (*p* < 0.05). Levels of FBG were declined in the LLY group than the HFFD group (*p* < 0.05). RSV treated mice had lower serum levels of lipids, FBG and LPS over the HFFD group (*p* < 0.05).

### 3.2. LY ameliorated NAFLD in the HFFD-fed mice

**Figure 2:**
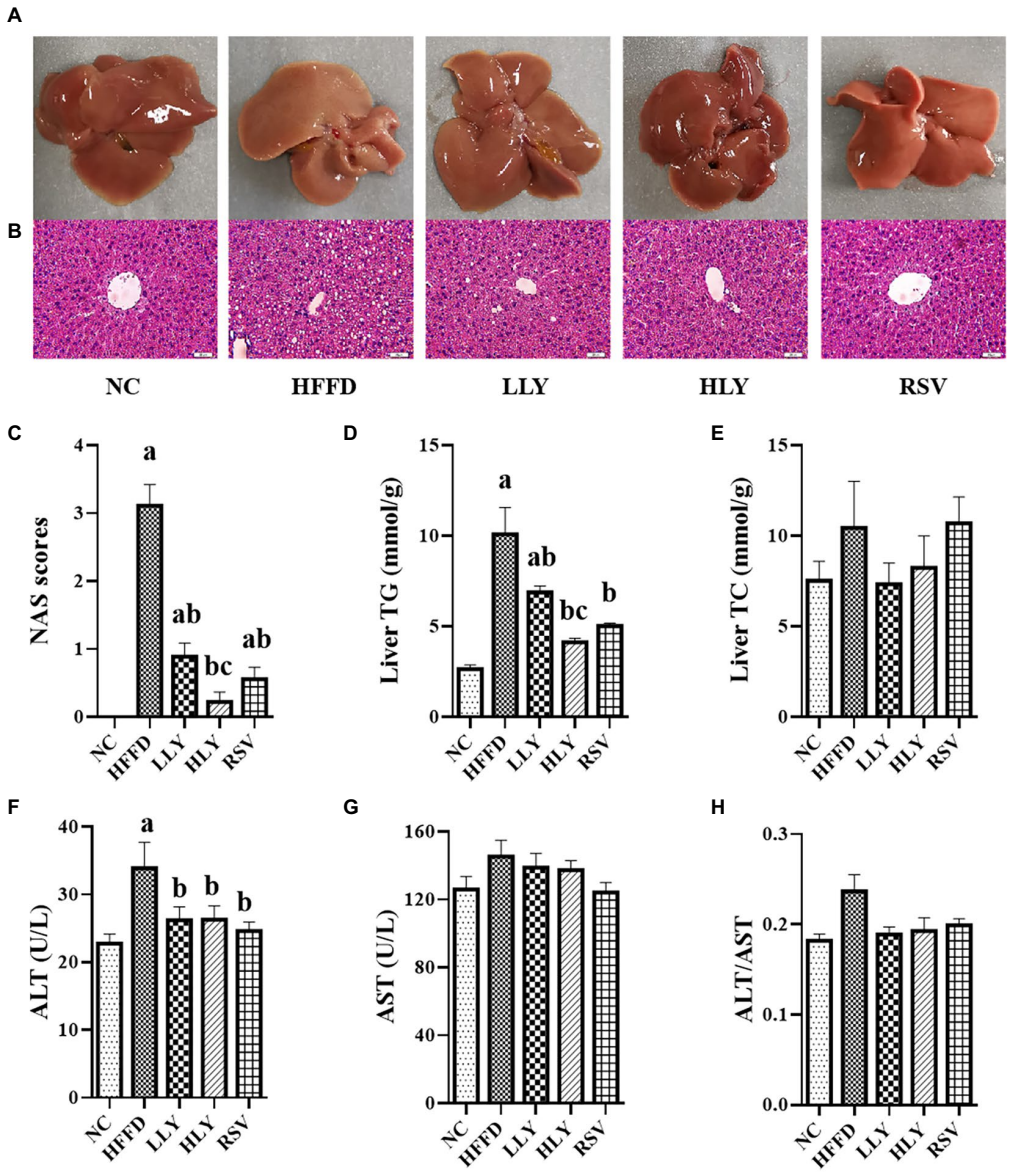
Effects of LY on the hepatic lipids and function. **(A)** General view of liver; **(B)** H&E staining of liver tissues (400×); **(C)** NAS scores; **(D)** Liver TG levels; **(E)** Liver TC levels; **(F)** Serum levels of ALT; **(G)** Serum levels of AST; **(H)** ALT/AST; ^a^*p* < 0.05, ^b^*p* < 0.05, ^c^*p* < 0.05 compared with the NC, HFFD and LLY groups, respectively.

Compared to the NC group, the livers of HFFD group were pale and rough by gross inspection, which were diminished by LY and RSV administration (Figure 2A). H&E staining of the livers revealed that the HFFD group displayed with severe steatosis and lipid droplet vacuoles accumulation (Figure 2B). NAS scores and concentrations of TG in liver of the mice in HFFD group were dramatically higher than the NC group (*p* < 0.05; Figures 2C,D). The hepatic steatosis was dramatically reduced in the LLY, HLY and RSV groups, accompany with lower NAS scores (*p* < 0.05) and hepatic TG levels (*p* < 0.05). In addition, the constituents of NAS scores, including steatosis scores, inflammation scores and balloning scores were all dramatically elevated in the HFFD group and notably ameliorated by LY and RSV (*p* < 0.05, Supplementary Table 2). The HLY group displayed the most notable attenuate effect. No significant change on hepatic TC levels was noticed among the groups (Figure 2E). In addition, HFFD feeding markedly enhanced serum levels of ALT (*p* < 0.05) and led to an increased trend of serum levels of AST and the ratio of ALT/AST (Figures 2F–H). LY and RSV administration tended to counteract these effects in the HFFD fed mice. Taken together, these results indicated that LY was capable of alleviating NALFD in mice.

### 3.3. Effects of LY on hepatic NLRP3 related inflammatory signaling pathway

**Figure 3:**
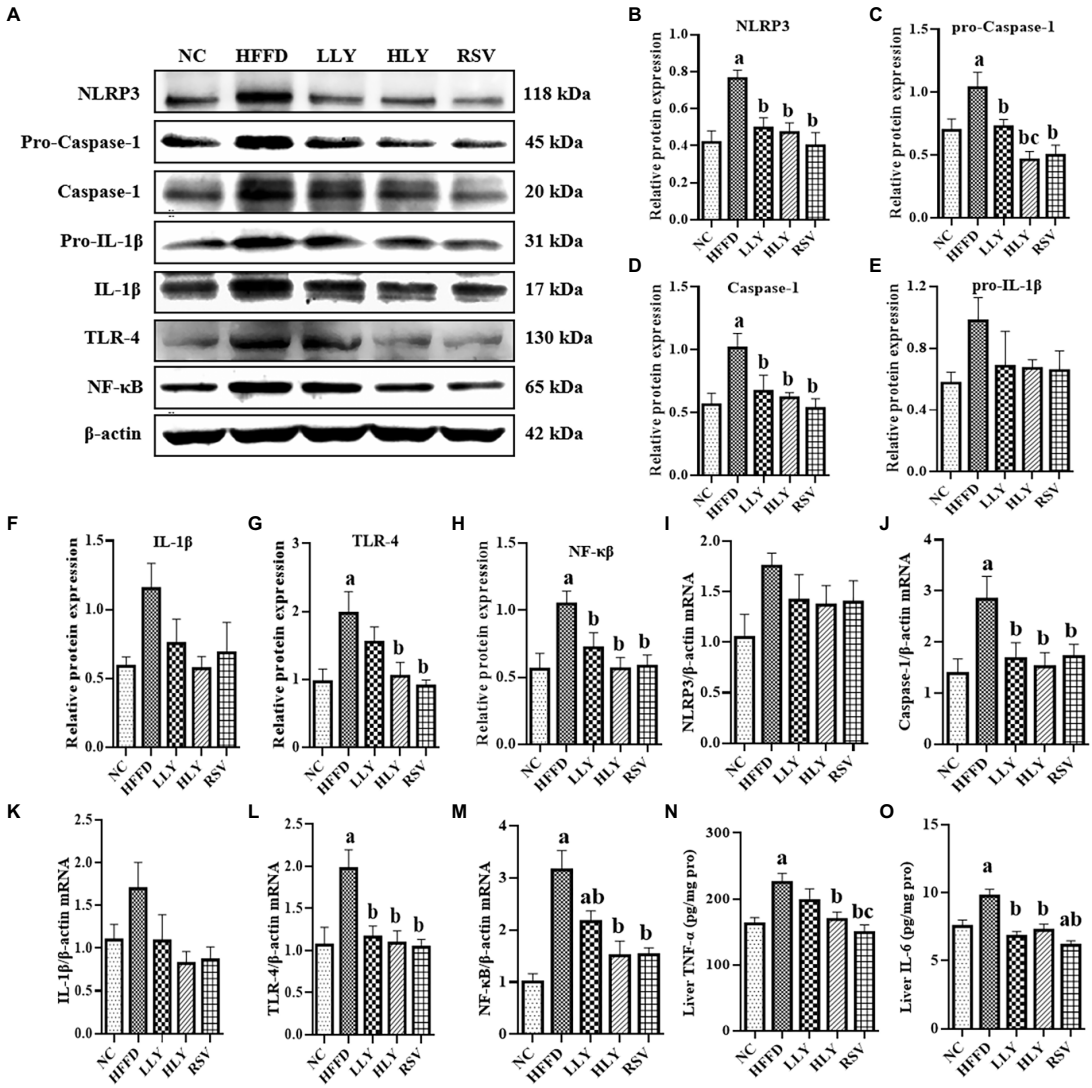
Effects of LY on NLRP3 related inflammatory signal pathway in liver. **(A)** Western blots of proteins in liver; serum TNF-α; **(B–H)** Relative protein expressions of NLRP3, pro-Caspse1, Caspse1, pro-IL-1β, IL-1β, TLR4 and NF-κB in liver; **(I–M)** Relative mRNA expression of NLRP3, Caspse1, IL-1β, TLR4 and NF-κB in liver; **(N)** Levels of TNF-α in liver; **(O)** Levels of IL-6 in liver; ^a^*p* < 0.05, ^b^*p* < 0.05, ^c^*p* < 0.05 compared with the NC, HFFD and LLY groups, respectively.

To explore the effect of LY on the hepatic NLRP3 inflammasome related inflammatory signaling pathway, we evaluated the expression of related genes in liver by western blot and RT-qPCR (Figure 3). Compared with the NC group, the protein levels of NLRP3, Pro-Caspase-1, Caspase-1, TLR4, NF-κB and the mRNA levels of Caspase-1, TLR-4, NF-κB were notably higher in the HFFD group (*p* < 0.05 for all), indicating the activation of the NLRP3 inflammasome related inflammatory pathway. LLY, HLY and RSV groups showed lower relative protein levels of NLRP3, Pro-Caspase-1, Caspase-1, NF-κB and the mRNA levels of Caspase-1, TLR-4, NF-κB in liver than the HFFD group (*p* < 0.05 for all). Relative protein expression of TLR4 was also lower in HLY and RSV groups than the HFFD group (*p* < 0.05). Although no significantly difference was observed on the protein levels of pro-IL-1β, IL-1β and mRNA levels of IL-1β, similar trend was observed.

We also measured the hepatic concentrations of TNF-α and IL-6. Elevated levels of IL-6 in the liver of HFFD-fed mice were significantly corrected by both LY and RSV (*p* < 0.05). High dose of LY and RSV also markedly diminished the enhanced levels of hepatic TNF-α in the mice of HFFD group.

### 3.4. The overall community structure of gut microbiota

**Figure 4:**
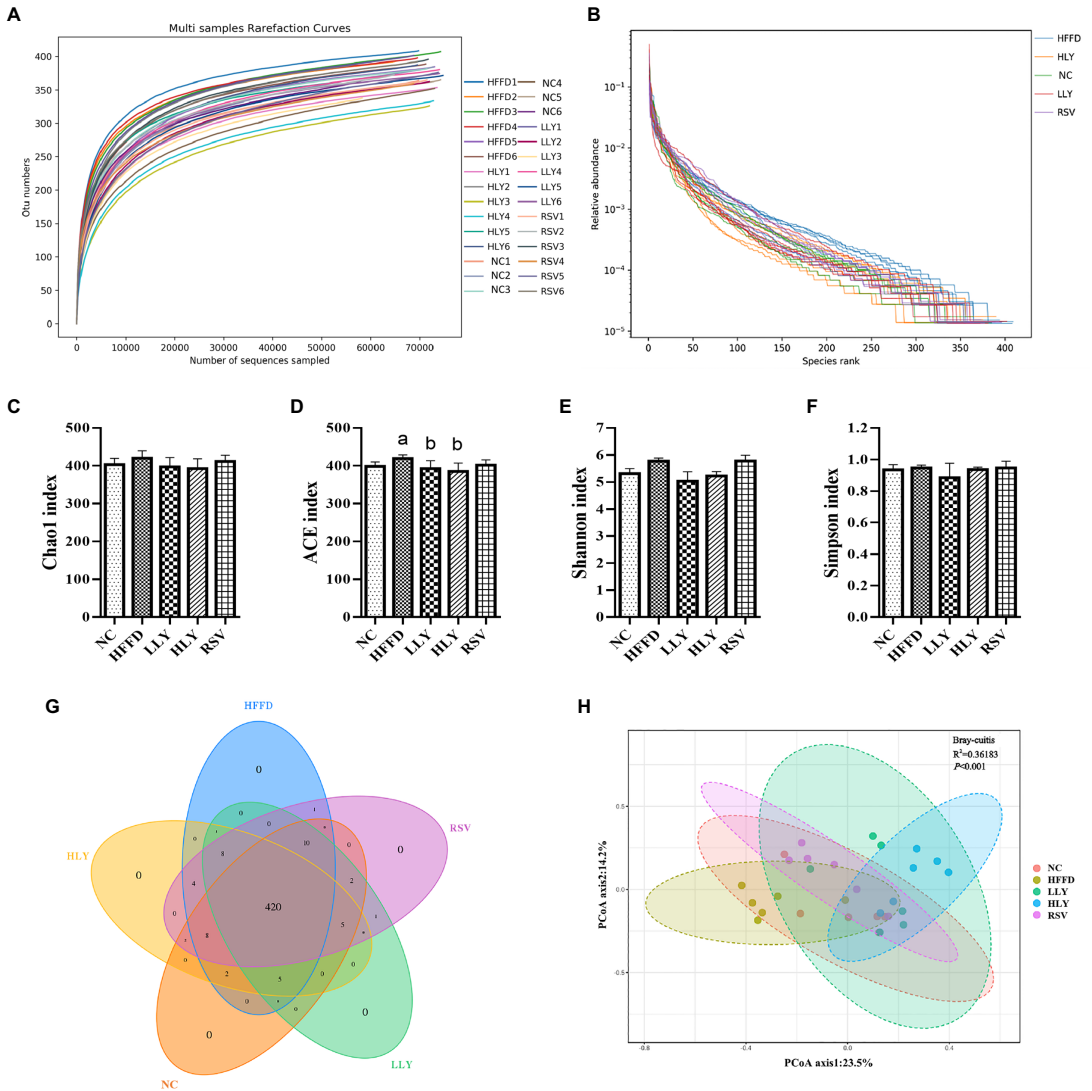
The overall community structure of fecal microbiota. **(A)** Rarefaction curves; **(B)** Rank abundance curves; **(C)** Chao 1 index; **(D)** ACE index; **(E)** Shannon index; **(F)** Simpson index; **(G)** Venn of the OTUs; **(H)** principal coordinates analysis. ^a^*p* < 0.05, ^b^*p* < 0.05, ^c^*p* < 0.05 compared with the NC, HFFD and LLY groups, respectively.

Alpha diversity reflects the diversity and richness of community. The flat dilution curves suggested sufficiency of sequencing depth (Figure 4A). Large span of the rank abundance curves on horizontal axis suggested high richness, and gentle trend of the curves on vertical axis reflected even distribution of the species (Figure 4B). Chao 1 and ACE indices determined the community richness, while Shannon and Simpson indices presented the diversity of the microbial communities (Figures 4C–F). The ACE index in HFFD group was higher than in NC group (*p* < 0.05) and ameliorated by the LY treatment (*p* < 0.05). No differences on Chao 1, Shannon and Simpson indices were observed among the experimental groups. The Venn of the OTUs (Figure 4G) showed 469 operational taxonomic units (OTUs) were obtained. Among them, the five groups were shared 420 OTUs. Collectively, these results indicated the similarity of the community richness and diversity among the different groups.

β-Diversity analysis performed *via* PCoA determined the structural differences of the fecal microbiota (Figure 4H). The experimental groups were well separated from each other, suggesting that the distinct microbial compositions of the mice in different groups.

### 3.5. LY ameliorated intestinal microbiota dysbiosis in the HFFD-fed mice

**Figure 5:**
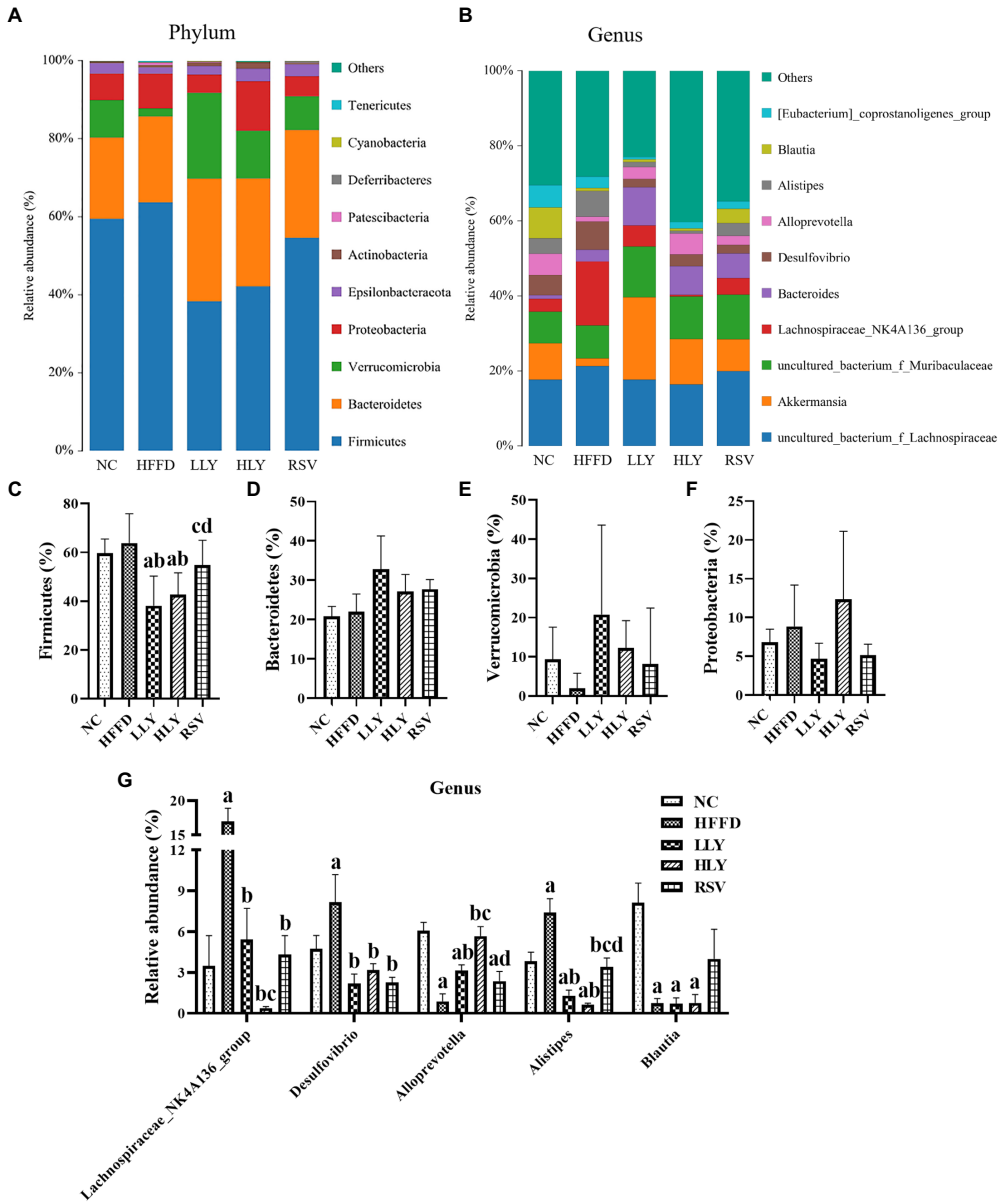
Gut microbiota composition among groups. **(A,B)** Microbial distributions at phylum and genus levels; **(C–F)** Relative abundance of bacteria at phylum levels; **(G)** Relative abundance of bacteria at genus levels. ^a^*p* < 0.05, ^b^*p* < 0.05, ^c^*p* < 0.05, ^d^*p* < 0.05 compared with the NC, HFFD, LLY and HLY groups, respectively.

Figures 5A,C–F shows the top 10 most abundant phyla of bacterial communities in the feces of the mice. Among which, *Firmicutes*, *Bacteroidetes*, *Verrucomicrobia*, and *Proteobacteria* taking approximately 95% of the total microbiota. HFFD diet feeding led to an increased trend of relative abundance of *Firmicutes*, *Proteobacteria*, and increased trend of *Verrucomicrobia*. Both low and high dose of LY groups displayed lower relative abundance of *Firmicutes* than the HFFD group (*p* < 0.05). The relative abundance of *Verrucomicrobia* had an increasing trend in the feces of LY and RSV treated mice.

The top 10 most abundant fecal genera are shown in Figures 5B,G. The relative levels of *Lachnospiraceae_NK4A136_group*, *Desulfovibrio*, and *Alistipes* were markedly higher, while those of *Alloprevotella* and *Blautia* were notably lower in the HFFD group than the NC group (*p* < 0.05). The bacterial genera of *Lachnospiraceae_NK4A136_group*, *Desulfovibrio*, and *Alistipes* were dramatically declined in the LLY, HLY and RSV groups than in the HFFD groups (*p* < 0.05). LLY and HLY groups also showed elevated levels of *Alloprevotella* than the HFFD group (*p* < 0.05).

To further clarify the roles of gut microbiota in regulation of NAFLD, the associations of LY modulated bacteria at genus levels with NAFLD-associated indices were also analyzed (Table 1). Fecal levels of *Lachnospiraceae_NK4A136_group* was positively correlated with serum lipids, LPS, liver IL-6, liver TG and mRNA levels of TLR4 and *NF-κB* (r: 0.375 to 0.582, *p* < 0.05). *Desulfovibrio* was positively associated with liver IL-6, TG and mRNA levels of *TLR4* and *NF-κB* (r: 367 to 0.435, *p* < 0.05). *Alloprevotella* was negatively related with serum lipids, FBG, INS, HOMA-IR, liver TG and mRNA levels of *NF-κB*, *NLRP3*, and *Caspase-1* (r: −0.379 to −0.607, *p* < 0.05). *Alistipes* was positively associated with mRNA levels of *TLR-4* in liver (r = 0.378, *p* < 0.05). *Blautia* was negatively related with serum TC, HDL-C, LDL-C, and liver TG (r: −0.396 to −0.543, *p* < 0.05) and positively associated with mRNA levels of *IL-1β* (r = 0.406, *p* < 0.05).

**Table 1:** Spearman correlation between gut microbiota at phylum and genus levels with NAFLD-related parameters.

	*Lachnospiraceae _NK4A136_group*	*Desulfovibrio*	*Alloprevotella*	*Alistipes*	*Blautia*
Serum ALT	0.355	0.051	−0.299	0.067	−0.098
Serum TG	0.375*	0.159	−0.485**	0.106	−0.241
Serum TC	0.403*	0.177	−0.572**	−0.033	−0.543**
Serum HDL-C	0.389*	0.240	−0.521**	−0.036	−0.507**
Serum LDL-C	0.466**	0.269	−0.605**	0.125	−0.396*
Serum FBG	0.065	0.336	−0.478**	0.094	−0.350
Serum INS	0.224	−0.029	−0.534**	0.052	−0.071
HOMA-IR	0.155	0.127	−0.607**	0.087	−0.212
Srum LPS	0.401*	0.092	−0.399	0.206	−0.096
Liver TG	0.232*	0.214*	−0.376**	0.081	−0.441*
Liver TNF-α	0.312	−0.018*	−0.210	0.141	−0.063
Liver IL-6	0.378*	0.424*	−0.120	0.334	−0.010
*TLR-4* mRNA	0.582**	0.435	−0.269	0.378*	−0.035
*NF-κB* mRNA	0.464**	0.334	−0.593**	0.211	−0.216
*NLRP3* mRNA	0.059	0.367*	−0.379*	0.219	0.143
*Caspase-1* mRNA	0.235	0.282	−0.586**	0.325	−0.079
*IL-1β* mRNA	0.124	0.305	0.133	0.162	0.406*

* *p* < 0.05;

** *p* < 0.01.

## 4. Discussion

In the current work, we evaluated the effect of LY on the hepatic NLRP3 inflammasome pathway and intestinal microbiota composition in NAFLD mice for the first time. LY dietary supplementation significantly prevented HFFD induced accumulation of fat in liver, decreased liver function, elevated serum levels of lipids and inflammatory cytokines, activation of hepatic NLRP3 inflammasome pathway and intestinal microbiota dysbiosis. The protective effect of NAFLD and hepatic inflammation by LY was also associated with the reshaped gut microbiota composition.

LY is a well-known natural product with notable antioxidant bioactivity and has tremendous potential to treat metabolic diseases. Herein, we found both low (20 mg/kg/d, equivalent to approximately 1.6 mg/kg for human) and high dose (60 mg/kg/d, equivalent to approximately 4.8 mg/kg for human) of LY supplementation remarkably reduced body weight gain, ameliorated serum dyslipidemia, decreased serum levels of ALT and inhibited hepatic TG accumulation in the HFFD -fed rats, indicating the protective effect of LY on NAFLD. Numerous studies reported the alleviative effect of LY (10–300 mg/kg/d) on hepatic steatosis, and dyslipidemia in high calorie diet (25) or chemicals (26) induced NALFD rodents, which are consistent with our findings. In addition, resveratrol, which is a natural polyphenol with notable anti-oxidant and anti-inflammation effects (27), was selected as a positive control in this study. In line with previous reports (28), our results also indicated that 50 mg/kg/d resveratrol dietary supplementation markedly prevented the development of NAFLD and the effects was comparable to high dose LY.

NAFLD is closely associated with hepatic chronic inflammation. NLRP3 inflammasome is essential for the progress of the chronic inflammation and has emerged as a potential new therapeutic target for NAFLD, recently (29). During the pathological process of NAFLD, TLR-4 in the surface of hepatocytes and macrophages of liver is activated by various damage or pathogen-activated molecular patterns (DAMPs or PAMPs), like LPS (9). Subsequently, the NF-κB inflammatory signaling pathway was activated to initiate NLRP3 inflammasome through promoting the expression of NLRP3, apoptosis-associated speck-like protein (ASC), pro-caspase-1, pro-IL-1β and pro-IL-18 (30). NLRP3 inflammasome is an intracellular multiprotein complex, which consists of NLRP3 receptor, an ASC adapter, and pro-caspase-1. The binding of NLRP3 to ASC promotes the maturation of caspase-1 to produce IL-1β/IL-18 from pro-IL-1β/IL-18, that aggravate hepatic inflammatory reaction and accelerate NAFLD development (31). In our study, we found that LY dramatically down-regulated the protein and mRNA expressions of TLR-4, NF-κB, NLRP3, Caspase-1, and IL-1β in the livers of NAFLD mice, indicating the inhibition of the NLRP3 inflammasome related pathway. The production of pro-inflammatory cytokines TNF-α and IL-6 in liver was also suppressed by LY, which further suggested the inhibition of hepatic inflammation. Zhu et al. reported that LY ameliorated atrazine-induced pyroptosis in spleen through blocking the NLRP3 inflammasome pathway in rodents (32). Another study proved that LY inhibited Di (2-ethylhexyl) Phthalate-induced Caspase-1, NLRP3, ASC, NF-κB, and IL-1β over-expression in spleen of male mice (33). Xue et al. showed that LY alleviated hepatic ischemia reperfusion injury *via* inhibition of NLRP3 inflammasome in Kupffer cells (34). These findings supported our results and our study was the first to clarify the suppression effect of LY on NLRP3 in the liver of NAFLD model.

Growing evidence indicates that cross-talk between the gut microbiota and liver is critical in the pathogenesis of NAFLD (35). Diet is the primary factor for the shaping of intestinal environment (36). In this study, the fecal microbial compositions were distinctly changed in the different diets-fed groups. Numerous specific gut bacteria exhibit important roles in the development of NAFLD. *Firmicutes* and *Bacteroidetes* are the most abundant bacterial phyla in the feces of both human and rodents (37). Various reports indicated that enhanced abundance of *Firmicutes* and declined that of *Bacteroidetes* were positively correlated to obesity, chronic inflammation status and NAFLD (38). At genus levels, *Lachnospiraceae_NK4A136_group* belongs to *Firmicutes* and is reported to be a discriminative feature of gut dysbiosis (39). *Desulfovibrio*, member of *Proteobacteria* phylum, is a typical pathogenic bacteria that produces LPS (40). *Alistipes* is a relatively new genus of pathogenic bacteria that is highly relevant in gut dysbiosis and metabolic diseases (41). *Allobaculum* and *Blautia* are SCFAs-producing microbiota (42), that are beneficial for protecting inflammation and NAFLD. In the present study, LY ameliorated intestinal microbiota dysbiosis through preventing the loss of beneficial bacteria, like *Allobaculum* and the increase of destructive bacteria, like *Firmicutes*, *Lachnospiraceae_NK4A136_group*, *Desulfovibrio*, and *Alistipes* in the NAFLD mice. Moreover, correlation analysis indicated that the LY regulated intestinal bacteria (negative for beneficial bacteria and positive for destructive bacteria) were all notably related with the NAFLD. LY is a fat-soluble component and mainly absorbed in small intestine through lymphatic system (43). During intragastric treatment, a large amount of un-absorbed LY will enter large intestine and influence gut environment (44). The protective effect of LY on gut ecosystem were also reported in other models of diseases, like obesity (45) and colitis (46). Our findings provided further evidence for the relationships among LY, gut microbiota and NLAFD. The gut-liver cross-talk was dependent on the microbial metabolites, mainly LPS and SCFA (10). LPS is a potent trigger of hepatic inflammation by binding to TLR-4 of liver and activating downstream NF-kB/ NLRP3 inflammasome pathway (10). LPS also destroys gut barrier, which facilitates the penetration of LPS into circulation. SCFAs are products of dietary fibers fermentation, that are beneficial for health. It is reported that SCFAs could promote the intestinal integrity and down-regulate TLR4-mediated inflammation (47). We found decreased serum levels of LPS, that is consistent with the results of intestinal flora. SCFAs-producing bacteria *Allobaculum* was restored by LY treatment as aforementioned. While, we failed to measure the fecal concentrations of SCFAs due to the limited feces acquired. Otherwise, in HFD-induced obese mice, dietary supplementation of 10 mg/kg LY significantly enhanced total cecal SCFAs concentrations (48). In an *in vitro* fermentation model, LY treatment also notably increased total SCFAs production of human gut flora (49). Collectively, we suppose the suppression of the NF-κB/NLRP3 inflammasome pathway in liver might be at least partially related to the modulation of intestinal microbiota and the microbial metabolites.

## 5. Conclusion

**Figure 6:**
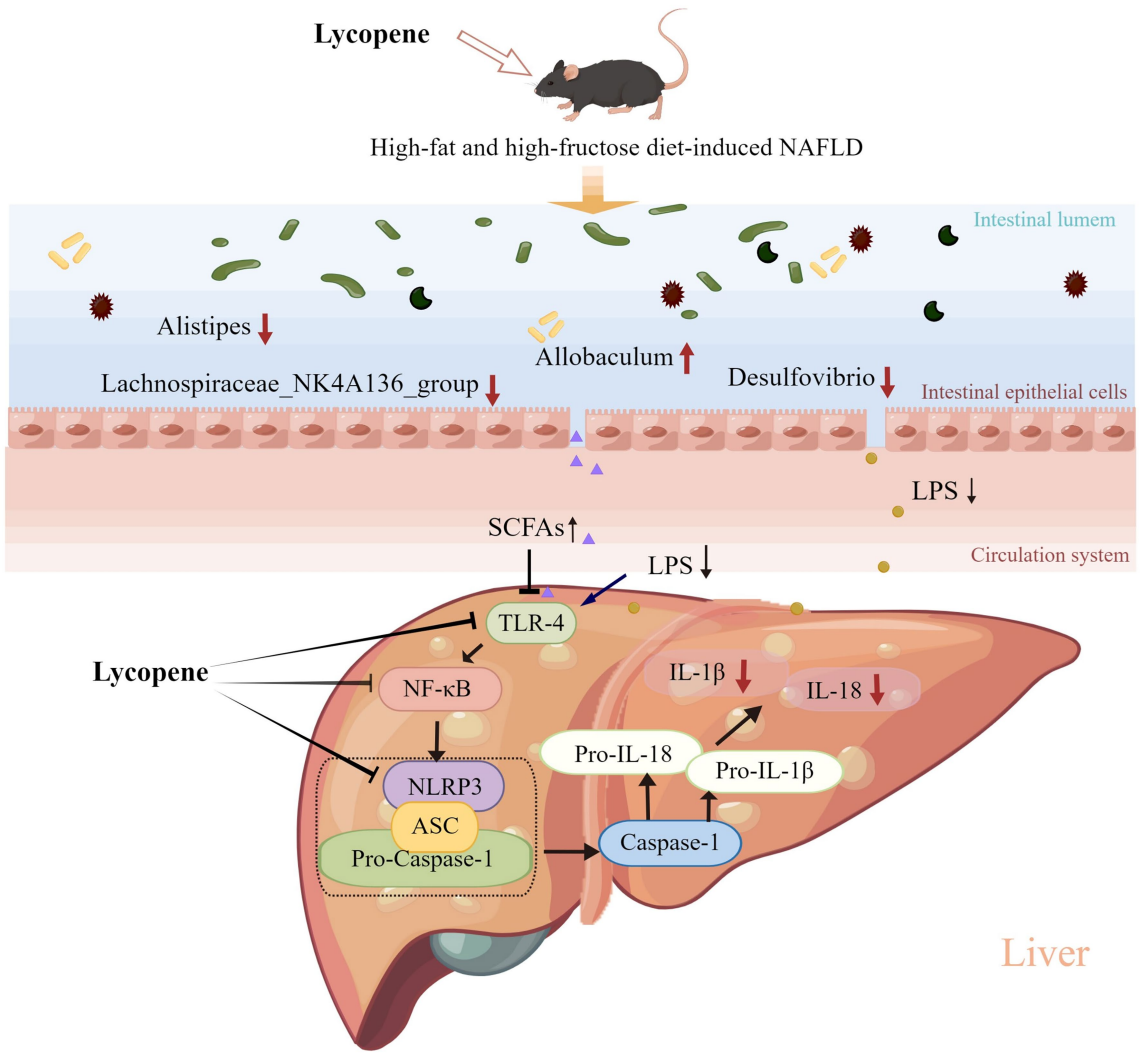
Potential mechanisms underlying the preventive effect of LY on HFFD-induced NAFLD. LY ameliorated HFFD-induced NAFLD through inhibition of hepatic TLR-4/NF-κB/NLRP3 inflammasome pathway and modulation of gut microbiota composition (decreasing LPS-producing bacteria and increasing SCFAs-producing bacteria).

In conclusion, our findings demonstrated that dietary LY supplementation ameliorated HFFD-induced NAFLD in mice and the potential mechanisms (Figure 6) were related to the inhibition of hepatic NF-κB/NLRP3 inflammasome pathway and modulation of gut microbiota composition. Our study provides a new insight into the mechanisms of LY in improving of NAFLD and support further evidence for the exploiting of LY as a functional ingredient for the prevention of NAFLD.

## Data availability statement

The original contributions presented in the study are publicly available. This data can be found at: https://www.ncbi.nlm.nih.gov/sra/PRJNA919496.

## Ethics statement

The animal study was reviewed and approved by the laboratory animal welfare ethics committee of Qingdao University.

## Author contributions

XG drafted the manuscript. HZ and XG performed the experiments and analyzed the data. XZ, ML, and YS revised the manuscript. YS designed the experiments and had primary responsibility for the final content of the manuscript. All authors contributed to the article and approved the submitted version.

## Funding

This work was supported by the National Natural Science Foundation of China (Nos. 81703206 and 81973015), Danone nutrition research and education fund (DIC 2019-09), and Science and Technology Program of Qingdao (No. 19-6-1-52-nsh).

## Conflict of interest

The authors declare that the research was conducted in the absence of any commercial or financial relationships that could be construed as a potential conflict of interest.

## Publisher’s note

All claims expressed in this article are solely those of the authors and do not necessarily represent those of their affiliated organizations, or those of the publisher, the editors and the reviewers. Any product that may be evaluated in this article, or claim that may be made by its manufacturer, is not guaranteed or endorsed by the publisher.

## Footnotes

^1^ https://www.microbiomeanalyst.ca./

^2^ https://international.biocloud.net

^3^ www.figdraw.com
