# Supplementary material for: Lycopene prevents non-alcoholic fatty liver disease through regulating hepatic NF-κB/NLRP3 inflammasome pathway and intestinal microbiota in mice fed with high-fat and high-fructose diet
Source: Front Nutr. 2023 Mar 23;10:1120254. doi: 10.3389/fnut.2023.1120254 (PMC10076551; doi:10.3389/fnut.2023.1120254)
Supplement: Supplementary file 1 [file Data_Sheet_1.docx]

Supplementary Material

Lycopene prevents non-alcoholic fatty liver disease through regulating hepatic NF-κB/NLRP3 inflammatory pathway and intestinal microbiota in mice fed with high-fat and high-fructose diet

[Xiang Gao](https://www.ncbi.nlm.nih.gov/pubmed/?term=Gao%20X%5bAuthor%5d&cauthor=true&cauthor_uid=31623137)^1,2^, Xia Zhao^3^, Min Liu^4^, Huimin Zhao^1^, [Yongye](https://www.ncbi.nlm.nih.gov/pubmed/?term=Li%20D%5bAuthor%5d&cauthor=true&cauthor_uid=31623137) Sun^1*^

*** Correspondence:** Corresponding Author: [yongye.sun@126.com](mailto:yongye.sun@126.com)

**Supplemental Table 1. Primers used during the real-time PCR assay.**

| Gene | Forward/reverse primer（5’-3’） |
| --- | --- |
| *TLR-4* | F: 5’-GGAAGTTCACATAGCTGAATGACAA-3’ |
|  | R: 5’-CAAGGCATGTCCAGAAATGAGA-3’ |
| *NF-κB* | F: 5’-CCCTCAAACGTCTGGACCTAA-3’ |
|  | R: 5’-AGCACAGCCTTCCTTGGTCA-3’ |
| *Caspase-1* | F: 5′-ACTCGTACACGTCTTGCCCTCA-3′ |
|  | R: 5′-CTGGGCAGGCAGCAAATTC-3′ |
| *NLRP3* | F: 5’-TCACAACTCGCCCAAGGAGGAA-3’ |
|  | R: 5’-AAGAGACCACGGCAGAAGCTAG-3’ |
| *IL-1β* | F: 5’-TCCAGGATGAGGACATGAGCAC -3’ |
|  | R: 5’-GAACGTCACACACCAGCAGGTTA -3’ |
| *β-actin* | F: 5’-TGCTGTCCCTGTATGCCTCTG -3’ |
|  | R: 5’-TGATGTCACGCACGATTTCC-3’ |

**Supplemental Table 2. Scores of steatosis, inflammation and balloning for calculating NAS scores.**

|  | NC | HFFD | LLY | HLY | RSV |
| --- | --- | --- | --- | --- | --- |
| Steatosis scores (0-3) | 0 | 2.11±0.20^a^ | 0.67±0.17^ab^ | 0.22±0.15^b^ | 0.56±0.18^ab^ |
| Inflammation scores (0-3) | 0 | 0.78±0.22^a^ | 0.22±0.15^b^ | 0^b^ | 0^b^ |
| Balloning scores (0-2) | 0 | 0.22±0.15^a^ | 0^b^ | 0^b^ | 0^b^ |
| NAS scores (sum) | 0 | 3.11±0.31^a^ | 0.89±0.20^ab^ | 0.22±0.15^bc^ | 0.56±0.18^ab^ |

Note: ^a^*p* < 0.05, ^b^*p* < 0.05, ^c^*p* < 0.05 compared with the NC, HFFD and LLY groups, respectively.
